# IL-5/IL-5R blockade enables safe non-steroidal anti-inflammatory agent reintroduction in non-steroidal anti-inflammatory exacerbated respiratory disease

**DOI:** 10.3389/fimmu.2026.1950229

**Published:** 2026-09-03

**Authors:** Pelin Korkmaz, Nevzat Kahveci, Semra Demir, Osman Ozan Yegit, Derya Unal, Merve Hormet Igde, Mehmet E. Sezgin, Ayse F. Aslan, Bircan Erden, Leyla Bolek, Merve D. Atasever, Selin E. Guzel, Şule Çelik Kamacı, Esra Kaya, Aslı Gelincik

**Affiliations:** Department of Internal Medicine, Division of Immunology and Allergic Diseases, Istanbul Faculty of Medicine, Istanbul University, Istanbul, Türkiye

**Keywords:** aspirin-induced asthma, benralizumab, mepolizumab, nasal polyps (NP), non-steroidal anti-inflammatory drugs (NSAIDs)

## Abstract

**Background:**

Biologic therapies targeting the interleukin-5 (IL-5) pathway improve asthma control and chronic rhinosinusitis with nasal polyposis (CRSwNP) in patients with non-steroidal anti-inflammatory drug (NSAID)-exacerbated respiratory disease (NERD). However, their effect on NSAID tolerance remains insufficiently characterized.

**Objective:**

This study aimed to investigate whether anti-IL-5/IL-5 receptor (IL-5R) treatments could improve NSAID tolerance in patients with NERD and Blended Reactions (BRs).

**Method:**

Clinical parameters, including the Asthma Control Test (ACT), 22-item Sino-Nasal Outcome Test (SNOT-22), visual analog scale (VAS) scores for quality of life, nasal polyp grade, and inhaled corticosteroid/long-acting β2-agonist (ICS/LABA) dose, were compared before and after biologic therapy. ASA provocation tests were repeated during biologic treatment and compared with prior challenge results; outcomes were also compared between the biologic-treated group and the biologic-naive comparison group.

**Results:**

Patients who received biological treatments showed significant improvements compared to baseline, with eosinophil counts decreasing from 695 (590–892.5) to 50 (30–60), ACT scores increasing from 12 (8.7–18) to 24 (20.7–24.2), SNOT-22 scores declining from 56 (51–73) to 16 (5.7–28.7), and VAS scores improving from 1 (0–2.2) to 9 (7–10), all with p=0.001. ASA provocation positivity decreased to 28.5% (p=0.002), and all patients stepped down from high-dose to medium/low-dose ICS/LABA. Among the four patients who remained intolerant, reactions occurred at higher cumulative doses, with delayed onset and milder symptoms.

**Conclusion:**

Our study indicated that blockade of IL-5 pathway significantly improved clinical outcomes and reduced ASA hypersensitivity in patients with NERD/BRs, highlighting their potential role in modifying disease phenotype beyond asthma control.

## Introduction

Non-steroidal anti-inflammatory drug (NSAID)-exacerbated respiratory disease (NERD) is a chronic eosinophilic airway disorder characterized by respiratory symptoms triggered by NSAID exposure, typically observed in individuals with asthma and/or chronic rhinosinusitis with nasal polyps (CRSwNP) (1). The reported prevalence of NERD varies according to population: it affects approximately 1.8% of the general population, increases to 7–15% in individuals with asthma, and is observed in up to one-third of patients with both asthma and CRSwNP (2, 3). Following NSAID ingestion, symptoms generally develop within 30 minutes and may range from rhinorrhea and nasal congestion to bronchospasm and respiratory distress (4). Notably, some patients present with simultaneous cutaneous and respiratory symptoms after NSAID intake, a pattern referred to as blended reactions (BRs), which do not fit neatly into existing European Network for Drug Allergy (ENDA)-EAACI categories due to the involvement of multiple organ systems (4, 5). The World Allergy Organization (WAO) statement has also acknowledged such mixed or overlapping presentations, emphasizing the need for broader categories that better capture the full clinical spectrum of NSAID hypersensitivity (6).

The severity and onset of NERD symptoms are thought to be linked to the degree of cyclooxygenase-1 (COX-1) inhibition, with weaker COX-1 inhibitors producing milder responses and selective cyclooxygenase-2 (COX-2) inhibitors rarely triggering exacerbations (1). In parallel, more than half of NERD patients show evidence of atopy, further supporting the role of eosinophilic and allergic inflammation in its pathophysiology (7, 8). Although the precise immunologic mechanisms remain partially uncharacterized, increasing evidence implicates intense type 2 eosinophilic inflammation and dysregulated eicosanoid metabolism, particularly through the overproduction of cysteinyl leukotrienes following COX-1 inhibition (9). Moreover, recent studies suggest that epigenetic modifications within arachidonic acid pathway genes may contribute to heightened inflammatory responses and NSAID susceptibility in this population (10).

Asthma is a clinically diverse condition that can manifest with varying phenotypes. Among these, eosinophilic inflammation is a hallmark feature of a specific subgroup typically characterized by late-onset, nonatopic asthma, often accompanied by CRSwNP (11). Beyond their role in promoting inflammation, eosinophils contribute directly to structural airway damage by releasing cytotoxic granule proteins and reactive oxygen species, which impair the epithelial barrier (12). Interleukin-5 (IL-5) not only facilitates eosinophil survival and activation but also enhances the release of profibrotic mediators such as transforming growth factor-beta (TGF-β), matrix metalloproteinases (MMPs), and platelet-derived growth factor (PDGF) from bronchial epithelial cells, perpetuating tissue damage (13). By targeting the IL-5/IL-5R pathway, both anti–IL-5 and anti–IL-5R monoclonal antibodies have been shown to reduce eosinophilic infiltration (13, 14).

Eosinophilic inflammation is a central driver in the pathophysiology of CRSwNP. Patients exhibiting high tissue eosinophil counts in polyp specimens frequently encounter more aggressive disease trajectories and elevated recurrence risks following surgical intervention (15). Anti–IL-5/IL-5R treatments such as mepolizumab have demonstrated effectiveness in reducing polyp size and improving sinonasal symptoms by suppressing eosinophilic inflammation, while benralizumab, targeting the IL-5 receptor alpha, has also shown significant efficacy by depleting both circulating and tissue eosinophils (16, 17).

Although type 2 biologics have emerged as promising options in the management of NERD, their impact on restoring NSAID tolerance remains controversial. While earlier case reports and small series suggested the development of tolerance to aspirin or NSAIDs in patients treated with agents such as omalizumab or dupilumab, subsequent real-world data have yielded inconsistent results (18–21). For instance, a pilot study demonstrated favorable outcomes regarding aspirin (ASA) provocation in patients receiving omalizumab and dupilumab, but not in those treated with mepolizumab or benralizumab (20). However, these findings were limited by small sample sizes and heterogeneous patient characteristics. This ongoing uncertainty highlights the need for further research, particularly in well-characterized patient subsets, to clarify the potential of biologics to achieve this goal as part of a personalized, patient-centered approach.

This study aimed to evaluate whether blockade of the anti–IL-5/IL-5R pathway improves NSAID tolerance in patients with NERD and BRs using ASA provocation testing performed as part of routine clinical assessment. By examining the relationship between eosinophilic suppression and NSAID reactivity, the study provides new insight into the potential impact of biologic agents on hypersensitivity profiles.

## Materials and methods

### Study population and baseline characteristics

This study included a total of 22 adult patients who were followed by the adult Division of Immunology and Allergic Diseases at Istanbul Faculty of Medicine. Fourteen patients had been initiated on anti–IL-5 or anti-IL-5R treatments (mepolizumab or benralizumab) due to severe asthma and CRSwNP, and eight patients who were not receiving biologic treatment were included as a biologic-naive comparison group. This group was not intended to serve as a matched control group, as patients receiving biologic therapy had more severe disease by indication. Rather, the biologic-naive group was included to provide a real-world reference for ASA reactivity in patients not receiving anti–IL-5/IL-5R therapy and to explore the possibility of spontaneous changes in ASA reactivity. All patients had a previously confirmed diagnosis of NSAID hypersensitivity established by a positive oral ASA provocation test performed before initiation of biologic therapy. Baseline ASA provocation test results obtained before initiation of anti-IL-5/IL-5R therapy were collected retrospectively from medical records. These challenges had been performed as part of routine clinical care in the same tertiary allergy center using a standardized oral ASA provocation protocol.

**Figure 1:**
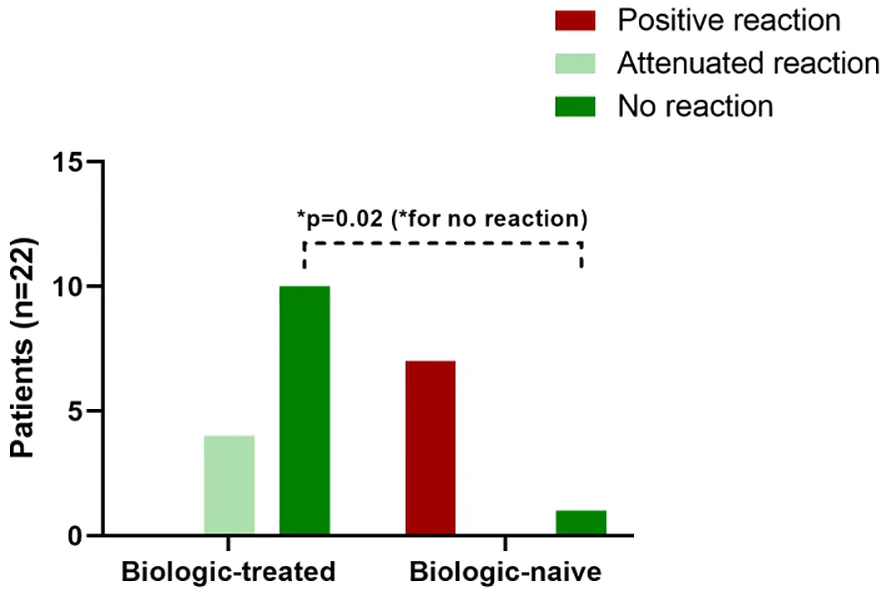
Study design and patient flow.

The Adult Division of Immunology and Allergic Diseases at Istanbul Faculty of Medicine is a tertiary referral center with expertise in advanced drug allergy evaluation. In our center, reassessment of NSAID hypersensitivity is considered as part of routine clinical practice in carefully selected patients with previously confirmed NSAID hypersensitivity who are under long-term follow-up, when reassessment is clinically appropriate, future NSAID use may become necessary, and the patient agrees to undergo repeated evaluation. Following ethics committee approval, these reassessments were prospectively documented within an observational framework. Repeated ASA provocation tests were performed using the same dosing schedule, monitoring procedures, and positivity criteria as those used for the baseline challenges. Written informed consent was obtained from all participants before enrollment (**Figure 1**).

At the time of current ASA provocation testing, a comprehensive set of demographic and clinical variables were documented. These included age, gender, smoking status, comorbid conditions, presence of chronic urticaria or other allergic diseases, and family history of allergy. Atopic status was assessed using skin prick testing (SPT) with a standard aeroallergen panel. Baseline pulmonary function was measured with spirometry immediately prior to provocation. Asthma diagnosis was confirmed in accordance with Global Initiative for Asthma (GINA) criteria, and data on the age at asthma onset and the current Inhaled Corticosteroid-Long-Acting Beta2-Agonist (ICS-LABA) dose according to GINA guidelines were recorded (22). Additional clinical information included history of allergic rhinitis, presence and age at onset of CRSwNP, number of endoscopic sinus surgeries (ESS), and current nasal polyp (NP) grading based on otorhinolaryngologist evaluation. The presence of other allergic comorbidities, such as food allergy, atopic dermatitis, or venom-induced anaphylaxis, was also recorded.

### ASA provocation test

The number and types of previous NSAID-induced hypersensitivity reactions experienced by each patient were documented in detail. The latency period between drug intake and the onset of symptoms were recorded in minutes.

During the ASA provocation test, all patients were closely monitored in a controlled clinical setting. Peripheral venous access was established, and baseline vital signs were obtained. Single-blind, placebo-controlled drug provocation tests were conducted under close hospital monitoring. Concomitant medications that could interfere with provocation testing, including antihistamines and beta-blockers, were discontinued prior to testing in accordance with guideline recommendations (23). ASA was administered in incremental oral doses of 50 mg, 100 mg, 150 mg, 250 mg, and 500 mg at 30-minute intervals, resulting in a cumulative dose of 1050 mg. These specific dose steps were selected based on the commercially available 100-mg and 500-mg ASA tablet formulations used in routine clinical practice in Türkiye. Although no universally standardized aspirin challenge protocol exists, the cumulative dose and incremental approach used in this study fall within the range of protocols described in the literature (24, 25). Patients were observed for at least 6 hours after the final dose, and delayed symptoms were assessed during follow-up within 72 hours. Vital signs were re-evaluated before each subsequent dose, and patients were continuously monitored for clinical symptoms. Spirometry was performed at baseline and repeated if respiratory complaints emerged during the procedure, regardless of symptom timing, and any decline in forced expiratory volume (FEV) was recorded. The drug provocation test was considered positive if the patient developed objective clinical signs such as a reduction in FEV in 1 Second (FEV_1_) of more than 20%, nasal symptoms (including sneezing, obstruction, or discharge), ocular manifestations like conjunctivitis, or cutaneous/systemic findings such as urticaria, angioedema, anaphylaxis, or maculopapular rashes following drug administration (26).

For each reaction, both the cumulative ASA dose and the exact time point (in minutes) at which symptoms developed were documented. Respiratory and cutaneous manifestations observed during the ASA provocation tests were carefully recorded. A ≥20% fall in FEV_1_ was defined as the provocative dose causing a 20% fall in FEV_1_ (PD20 FEV_1_) threshold, and the time and cumulative dose associated with this decline were noted. Reactions during the NSAID provocation were classified into three categories: positive reaction, attenuated reaction, and no reaction.

### Evaluation of anti–IL-5/IL-5R treatment responses

Pre-treatment variables including peripheral eosinophil counts, total immunoglobulin E (IgE) levels, Asthma Control Test (ACT) scores, Sino-Nasal Outcome Test (SNOT-22) scores, visual analog scale (VAS) scores for quality of life (0 = worst perceived status, 10 = best), NP grading, and GINA step classification of asthma treatment were retrieved from medical records for all patients who received biologics (27–30). These parameters were subsequently compared with the current status assessed during the ASA provocation following biologic treatment. Comparable evaluations were also conducted in the biologic-naive comparison group, allowing for intergroup comparisons. Additionally, the specific biologic agent administered (mepolizumab or benralizumab), any prior treatment with omalizumab, the biologic dose number at which the ASA provocation test was performed, and the time interval (in days) between the last biologic administration and the ASA provocation were all recorded. Because this was a real-world observational study, repeat ASA provocation testing was not performed at a predefined treatment time point. Instead, patients underwent reassessment after varying durations of biologic therapy according to routine clinical follow-up and patient consent.

### Statistical analysis

Baseline demographic and clinical characteristics were compared between patients receiving anti-IL-5/IL-5R treatment and biologic-naive patients. Clinical outcomes before and after biologic treatment were evaluated within the biologic-treated group. In addition, biologic-treated patients were stratified according to the outcome of the repeat ASA provocation test (ASA-tolerant versus ASA-reactive), and subgroup analyses were performed to explore factors associated with persistent ASA reactivity. Descriptive statistics such as continuous variables were presented as median and interquartile range (IQR) due to the small number of patients, while categorical variables were expressed as frequencies and percentages. For group comparisons, the Mann–Whitney U test was used for continuous variables, while the chi-square test or Fisher’s exact test was applied for categorical variables. Paired comparisons of continuous variables before and after biologic therapy were performed using the Wilcoxon signed-rank test. Changes in categorical variables (e.g., ICS–LABA dose before and after biologic therapy, ASA provocation test results) were analyzed using McNemar’s exact test.

A two-sided p-value of less than 0.05 was considered statistically significant. Statistical analyses were conducted using IBM SPSS Statistics for Windows, Version 22.0 (IBM Corp., Armonk, NY). Figures were created with GraphPad Prism 9 (GraphPad Software, San Diego, CA).

### Ethical consideration

The study was approved by the Ethics Committee (Institutional Review Board) of Istanbul Faculty of Medicine and conducted in accordance with the Declaration of Helsinki (Approval No. 11.09.2025-3580096).

## Results

### Comparison of baseline clinical findings between biologic-treated and biologic-naive patients

Baseline demographic and clinical characteristics of patients receiving biologic treatment (n=14) and those without biologic therapy (n=8) are summarized in **Table 1**. No significant differences were observed between the groups in terms of age, gender, smoking status, comorbidities, or NP severity. Lung function parameters and serum total IgE levels were also comparable. Patients without biologic treatment exhibited significantly higher peripheral eosinophil counts (p=0.002) and higher ACT scores (p=0.02). Also, patients receiving biologic treatment exhibited significantly higher VAS scores for quality of life compared to those without biologic treatment (p = 0.04). The proportion of NERD/BRs was 12 (85.7%)/2 (14.3%) in the biologic-treated group and 5 (62.5%)/3 (37.5%) in the biologic-naive group (**Table 2**).

**Table 1:** Demographic and clinical data of patients.

Characteristics	Patients receiving mepolizumab or benralizumab (n = 14)	Patients not receiving biological treatment (n = 8)	p-value
*Age, median (IQR)*	36.5 (29.5-50.5)	43.5 (31.5-51)	NS
*Female sex, n (%)*	8 (57.1)	2 (25)	NS
*Smoking status (Current/Former/Never)*	1/3/10	1/2/5	NS
*Comorbidity, n (%)*	2 (14.2)	2 (25)	NS
*Hypertension*	0 (0)	1 (12.5)	
*CSU*	1 (7.1)	1 (12.5)	
*Coronary artery disease*	1 (7.1)	0 (0)	
*Personal history of allergic conditions, n (%)*	9 (64.3)	4 (50)	NS
*Allergic rhinitis*	9 (64.3)	3 (37.5)	NS
*Drug allergy (other than NSAID)*	1 (7.1)	1 (12.5)	NS

*CSU, Chronic Spontaneous Urticaria, NS, Not significant.*

**Table 2:** Clinical, functional, and biohumoral data at the time of ASA provocation testing.

Characteristics	Patients receiving mepolizumab or benralizumab (n = 14)	Patients not receiving biological treatment (n = 8)	p-value
*NERD/BRs, n (%)*	12 (85.7)/2 (14.3)	5 (62.5)/3 (37.5)	NS
*Age at first NSAID reaction, median (IQR)*	25.5 (20.7-35.7)	29 (18-41.7)	NS
*Number of NSAIDs with reactions, median (IQR)*	4 (3-7)	3 (2-8.7)	NS
*Most frequent NSAID group, n (%)*			NS
*Salicylic acid derivatives*	14 (100)	8 (100)	NS
*Aryl‐propionic acids*	12 (85.7)	8 (100)	NS
*Hetero‐aryl acetic acids*	7 (50)	3 (37.5)	NS
*Pyrazolone derivatives*	4 (28.6)	2 (25)	NS
*Enolic acids*	2 (14.3)	1 (12.5)	NS
*Positivity to at least one inhalant allergen, n (%)*	6 (42.9)	3 (37.5)	NS
*HDM*	6 (42.9)	3 (37.5)	
*Weed pollen*	1 (7.1)	0 (0)	
*Grass pollen*	1 (7.1)	0 (0)	
*Tree pollen*	1 (7.1)	0 (0)	
*Grass-cereal mix*	1 (7.1)	0 (0)	
*Alternaria*	1 (7.1)	0 (0)	
*Cat dander*	1 (7.1)	0 (0)	
*Asthma*	14 (100)	8 (100)	NS
*Age at asthma diagnosis (years), median (IQR)*	23.5 (17.7-31)	24 (17.2-36.7)	NS
*CRSwNP, n (%)*	12 (85.7)	5 (62.5)	NS
*Age at CRSwNP diagnosis (years), median (IQR)*	23.5 (20-30)	18 (16.5-42.5)	NS
*Number of ESS, median (IQR)*	2 (1-4.7)	2 (1-4)	NS
*NP Grade (maximum score from either side, during ASA provocation)*			NS
*No NP (post-surgery)*	5 (35.7)	2 (25)	NS
*Grade 1*	2 (14.3)	1 (12.5)	NS
*Grade 2*	1 (7.1)	2 (25)	NS
*Grade 3*	2 (14.3)	0 (0)	NS
*Grade 4*	2 (14.3)	0 (0)	NS
			
*Family history of allergic conditions, n (%)*	6 (42.9)	3 (37.5)	NS
*Asthma, n (%)*	3 (21.4)	0 (0)	
*Allergic rhinitis, n (%)*	4 (28.6)	2 (25)	
*Atopic dermatitis, n (%)*	0 (0)	0 (0)	
*Venom allergy, n (%)*	0 (0)	1 (12.5)	
*Food allergy, n (%)*	0 (0)	0 (0)	
*Drug allergy, n (%)*	1 (7.1)	0 (0)	
*FEV_1_/FVC ratio, median (IQR), during ASA provocation*	75.56 (71.8-82.8)	77.7 (70.4-83.1)	NS
*FVC liter, median (IQR), during ASA provocation*	4.15 (3.8-4.3)	4 (3.9-4.7)	NS
*FVC (% predicted), median (IQR), during ASA provocation*	102.7 (90.5-114)	105.2 (96.6-117.3)	NS
*FEV_1_ liter, median (IQR), during ASA provocation*	3.1 (2.8-3.3)	3.2 (3-3.4)	NS
*FEV_1_ (% predicted), median (IQR), during ASA provocation*	91.6 (81.4-103.5)	97.8 (93.8-109.9)	NS
*Serum total IgE, median (IQR), IU/mL, during ASA provocation*	171.8 (67.7-288.7)	117.3 (52.1-170.4)	NS
*Peripheral EO count, median (IQR), cells/μL, during ASA provocation*	50 (30-60)	220 (145-407.5)	p=0.002
*ACT, median (IQR), during ASA provocation*	24 (20.7-24.2)	25 (24.2-25)	p=0.02
*SNOT-22, median (IQR), during ASA provocation*	16 (5.7-28.7)	17 (7.5-25.7)	NS
*VAS score for quality of life, median (IQR), during ASA provocation*	9 (7.8-10)	7 (6-7.8)	p=0.04

NERD, NSAID-Exacerbated Respiratory Disease, BRs, Blended Reactions, NSAID, Nonsteroidal Anti-Inflammatory Drug, HDM, House Dust Mite, NP, Nasal Polyposis, ESS, Endoscopic Sinus Surgery, FEV_1_, Forced Expiratory Volume in 1 Second, FVC, Forced Vital Capacity, EO, Eosinophil, ACT, Asthma Control Test, SNOT-22, Sino-Nasal Outcome Test-22, VAS, Visual Analog Scale, ICS, Inhaled Corticosteroid, LABA, Long-Acting Beta2-Agonist, NS, Not significant.

Bold values indicate statistical significance (p < 0.05).

Baseline characteristics of patients receiving anti–IL-5/IL-5R biologics are shown in Supplement 1 in detail. Four patients had received prior omalizumab treatment, which was discontinued due to lack of clinical response. Mepolizumab was the most frequently prescribed biologic (n=11), while 3 patients received benralizumab. The median age at biologic initiation was 34 years, and all 14 patients were treated with biologic treatment for severe asthma. The median number of previous ESS procedures was 2 (1–6). Concurrent ESS during biologic treatment was performed in six patients, all within the first month after biologic initiation. Postoperative healing was uneventful, and no recurrence of nasal polyps was observed in these patients during the available postoperative follow-up. Based on prior ASA provocation, 12 patients had NERD and two had BRs (**Supplementary 1**).

Baseline characteristics of biologic-naive patients are summarized in **Supplementary Table Supplement 2**.

### ASA provocation results in biologic-treated and biologic-naive patients

**Figure 2:**
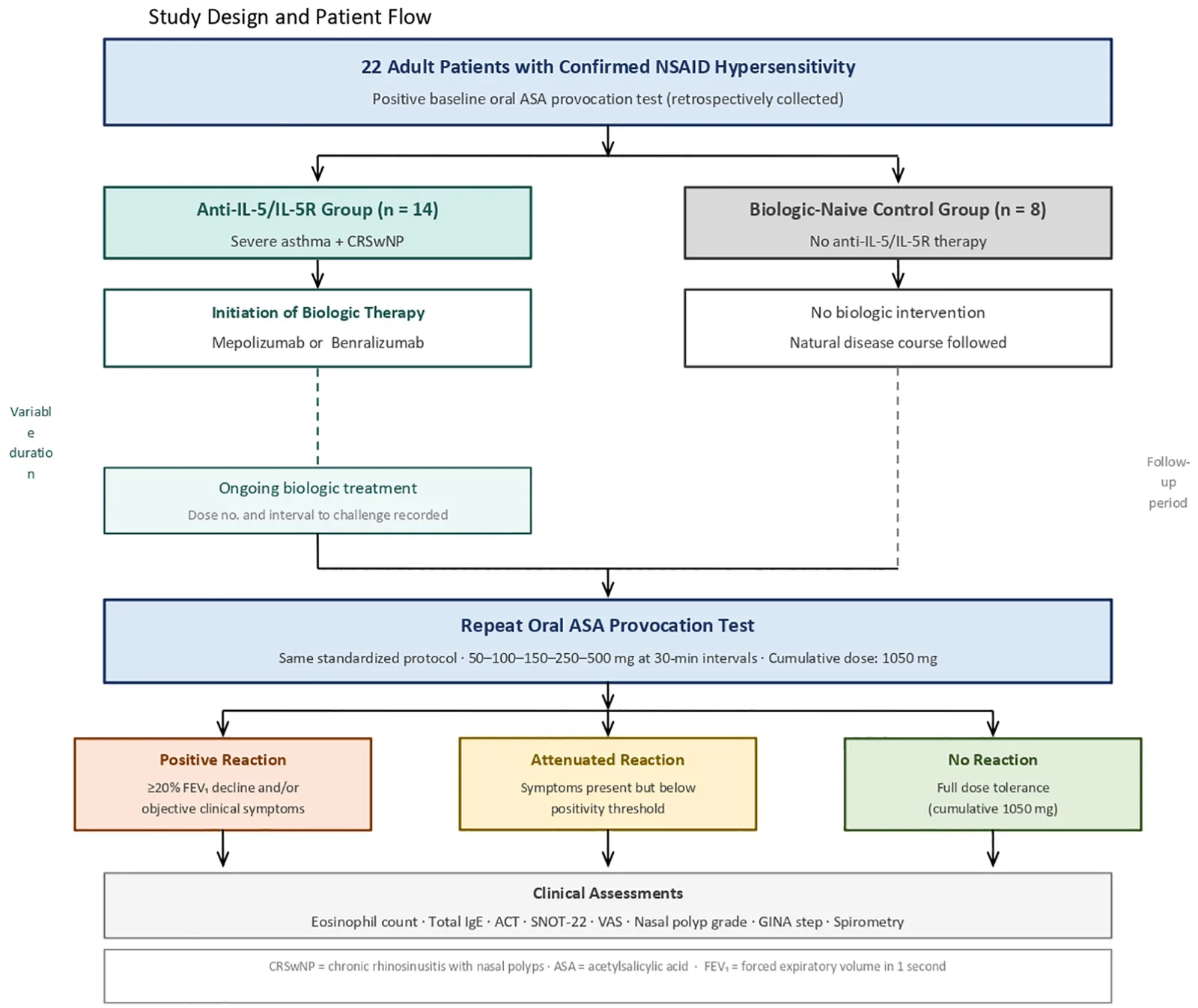
Proportion of patients with complete ASA tolerance in biologic-treated vs non-treated groups.

Among patients receiving biologic therapy (mepolizumab or benralizumab) (n=14), positive ASA reactions were significantly less frequent compared with those not receiving biologic treatment (28.6% vs. 87.5%, p=0.02) (**Figure 2**, **Supplement 1**). The median time interval between drug intake and reaction onset did not differ significantly between groups [98.5 minutes (IQR 62.7–123) vs. 135 minutes (IQR 105–190); p>0.05]. Similarly, the cumulative ASA dose at the time of reaction was comparable [850 mg (IQR 387.5–1050) vs. 1050 mg (IQR 1050–1050); p>0.05]. Among eight patients not receiving biologic therapy, one patient experienced spontaneous remission within eight years (**Figure 2**, **Supplementary Table Supplement 2**).

**Figure 3:**
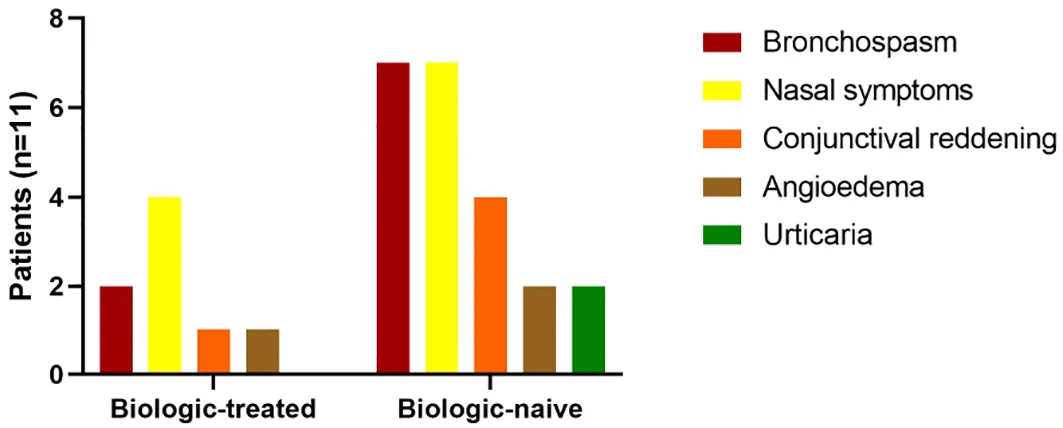
Clinical features of positive ASA reactions in biologic-treated and non-treated patients.

The frequency and distribution of clinical findings among patients with positive ASA reactions are presented in **Figure 3**. When the analysis was restricted to patients with positive ASA reactions (n=11), the distribution of bronchospasm, nasal, conjunctival, angioedema, and urticaria symptoms did not differ significantly between biologic-treated (n=4) and non-treated (n=7) groups (all p > 0.05) (**Figure 3**).

### Clinical and laboratory outcomes in biologic-treated patients

At the time of ASA provocation testing, patients had received anti-IL-5/IL-5R treatment for a median duration of 13.5 months (IQR 3–40 months). Following biologic treatment, patients demonstrated a marked reduction in peripheral eosinophil counts (p=0.001), alongside significant improvements in ACT scores (p=0.001), SNOT-22 (p=0.001), and VAS quality-of-life scores (p=0.001). ASA provocation positivity decreased substantially (p=0.002). In addition, all patients were able to step down from high-dose ICS/LABA therapy, with 100% maintained on medium- or low-dose treatment (p<0.001 (**Table 3**).

**Table 3:** Clinical and functional outcomes before and after anti-IL-5/IL-5R treatment.

Outcome	*Before biological treatment*	*After biological treatment*	*p-value*
*EO (cells/μL), median (IQR)*	*695 (590-892.5)*	*50 (30-60)*	*p=0.001*
*ACT score, median (IQR)*	*12 (8.7-18)*	*24 (20.7-24.2)*	*p=0.001*
*SNOT-22, median (IQR)*	*56 (51-73)*	*16 (5.7-28.7)*	*p=0.001*
*VAS QoL, median (IQR)*	*1 (0-2.2)*	*9 (7-10)*	*p=0.001*
*ASA provocation positivity, n (%)*	*14 (100)*	*4 (28.5)*	*p=0.002*
ICS-LABA dose			
*High-dose (n (%))*	*12 (85.8%)*	*0 (0%)*	*p<0.001*
*Low-/Medium-dose (n (%))*	*2 (14.2%)*	*14 (100%)*	

ASA, Acetylsalicylic Acid, EO, Eosinophil, ACT, Asthma Control Test, SNOT-22, Sino-Nasal Outcome Test-22, VAS, Visual Analog Scale, QoL, Quality of Life, ICS, Inhaled Corticosteroid, LABA, Long-Acting Beta2-Agonist.

When biologic-treated patients were stratified into ASA-tolerant (n=10) and ASA-reactive (n=4) groups, no statistically significant differences were observed in demographic or clinical characteristics, including age, gender, atopy status, baseline total IgE levels, age at biologic initiation, concomitant endoscopic sinus surgery, duration of biologic treatment, or the interval between the last biologic dose and ASA provocation testing (**Table 4**).

**Table 4:** Comparison of ASA-tolerant and ASA-reactive patients following anti-IL-5/IL-5R treatment.

Characteristics	Biologic: ASA-tolerant (n=10)	Biologic: ASA-reactive (n=4)	*p-value*
*Age, median (IQR)*	46 (32.5-52.5)	30 (26.2-30.7)	NS
*Female sex, n (%)*	5 (50)	3 (75)	NS
*Atopy, n (%)*	3 (30)	3 (75)	NS
*Baseline total IgE (IU/mL), median (IQR)*	176 (38.7-345.5)	171.8 (117.6-219.1)	NS
*Age at biologic initiation (Anti-IL-5/IL-5R), median (IQR)*	43 (31.7-49)	28.5 (25.5-30.7)	NS
*Concomitant ESS with biologic therapy, n (%)*	4 (40)	1 (25)	NS
*n° of biologic at the time of ASA provocation test, median (IQR)*	19 (4.5-48)	4 (3-31.2)	NS
*ASA provocation interval (number of days after last dose of biologic), median (IQR)*	16.5 (5.7-20.7)	13.5 (5.5-14)	NS

ASA, Acetylsalicylic Acid; lL5, Interleukin-5; ESS, Endoscopic Sinus Surgery; n°, number; NS, Not significant.

Detailed outcomes of the four biologic-treated patients who remained ASA-reactive are presented in **Table 5**. Compared with their previous provocation results, all four patients tolerated higher cumulative ASA doses with delayed reaction times. Moreover, the reaction phenotype shifted toward milder manifestations, most frequently limited to nasal or conjunctival symptoms. Only one patient (P-12) continued to experience bronchospasm, albeit at a higher ASA dose and with a later onset. Importantly, at baseline, the initial reaction doses and times did not differ significantly between these four patients and the ten who subsequently became ASA-tolerant (**Table 5**).

**Table 5:** ASA provocation before vs after anti-IL-5/IL-5R in ASA-reactive patients.

Patient ID	Biologic agent	Previous ASA reaction dose (mg)	Previous ASA reaction type	Time to previous ASA reaction (min)	Current ASA reaction dose (mg)	Current ASA reaction type	Time to current ASA reaction (min)
P-4	Benralizumab	300	Bronchospasm, nasal symptoms, conjunctival erythema	65	1050	Nasal symptoms	145
P-7	Mepolizumab	150	Bronchospasm, nasal symptoms, conjunctival erythema	40	300	Dyspnea, nasal symptoms	67
P-9	Mepolizumab	50	Bronchospasm, nasal symptoms, conjunctival erythema	15	1050	Nasal symptoms	137
P-12	Benralizumab	150	Bronchospasm, nasal symptoms, conjunctival erythema	42	550	Bronchospasm, nasal symptoms, conjunctival erythema	105

ASA, Acetylsalicylic Acid; Il-5, Interleukin-.5

## Discussion

In this study, we demonstrated that blockade of IL-5/IL-5R pathway markedly reduced ASA provocation positivity from 100% to 28.5%. Even among the four patients who remained reactive, reactions occurred at higher cumulative ASA doses, with delayed onset and milder symptoms, indicating a clear attenuation of hypersensitivity. Beyond this pivotal effect, anti–IL-5/IL-5R therapies also improved asthma-related outcomes, with significant reductions in eosinophil counts, improved ACT, SNOT-22, and VAS scores, and facilitated step-down from high-dose ICS/LABA therapy in all patients. To our knowledge, this is the first study to prospectively evaluate biologic effects in a study including both NERD and BRs. Unlike previous reports limited to 6-month evaluations, we additionally assessed patients at the third biologic dose, providing an early insight into tolerance development. Collectively, these findings suggest that anti-IL-5/IL-5R biologics may exert disease-modifying effects beyond asthma control in NERD and BRs.

Sánchez et al. recently conducted a randomized comparative trial evaluating different biologics in NERD and reported NSAID tolerance in 22% of patients treated with mepolizumab and 22% of those treated with benralizumab; however, unlike omalizumab and dupilumab, neither anti-eosinophilic treatment was associated with a statistically significant increase in ASA tolerance (20). In contrast, 71.4% of patients receiving anti-IL-5/IL-5R therapy in our cohort tolerated ASA during repeat provocation. Several methodological and population-related differences may account for this discrepancy. Sánchez et al. evaluated relatively small individual biologic groups at a fixed six-month time point and used an ASA challenge protocol with a cumulative dose of up to 925 mg, whereas our study evaluated anti-IL-5/IL-5R therapy as a therapeutic class in a real-world cohort, with repeat ASA provocation performed after varying durations of biologic exposure. In addition, our population included patients with blended reactions as well as NERD, which may further limit direct comparison between the two studies. Given the small sample sizes in both studies and these differences in study design and patient characteristics, the apparently higher tolerance rate observed in our cohort should therefore be interpreted cautiously and requires confirmation in larger prospective studies. Importantly, tolerance development was not dependent on the timing of the last biologic dose, a novel observation that needs confirmation by more comprehensive studies. Taken together, these findings indicate that anti–IL-5/IL-5R therapy can substantially modulate ASA reactivity, even in patients who remain clinically reactive. Although these four patients continued to exhibit ASA-induced symptoms, their reactions were consistently milder, occurred at higher cumulative doses, and were delayed in onset compared with baseline provocations. This suggests that anti–IL-5/IL-5R therapies may still exert clinically meaningful effects short of complete tolerance. Importantly, because tolerance development may emerge over subsequent cumulative doses, even in initially reactive patients, future follow-up could reveal further improvement.

The pathophysiology of NERD involves a redirection of arachidonic acid metabolism toward the 5-lipoxygenase pathway following COX-1 inhibition, leading to excessive cysteinyl leukotriene production by mast cells, basophils, and particularly eosinophils (24). LTE_4_ amplifies mast-cell activation and airway inflammation, sustaining the eosinophilic and type-2 inflammatory response characteristic of NERD. By depleting eosinophils and disrupting the eosinophil–mast-cell interaction, anti-IL-5/IL-5R therapy reduces cysteinyl leukotriene production and interrupts this amplification loop (31). Accordingly, the improved ASA tolerance observed in patients receiving anti-IL-5/IL-5R therapy is biologically plausible within this framework. It should also be noted that the biologic-naive comparison group consisted of patients with well-controlled asthma who did not meet the indication criteria for biologic therapy; therefore, the absence of anti-IL-5/IL-5R treatment in this group reflects clinical appropriateness rather than selection bias. Interestingly, one patient showed improved NSAID tolerance even before the initiation of biologic therapy, suggesting that individual variability in disease activity may occasionally influence clinical outcomes. Nevertheless, this isolated observation should be interpreted with caution, as NERD is generally considered a persistent condition (1).

Moreover, skin symptoms such as urticaria and angio-oedema are increasingly recognized as relevant comorbidities in the spectrum of NERD (32). While the classic triad focuses on asthma, nasal polyposis and NSAID intolerance, a subset of patients may also exhibit cutaneous manifestations. In the absence of concomitant chronic spontaneous urticaria, this coexistence is more appropriately referred to as a “blended phenotype” rather than a true overlap with aspirin/NSAID-exacerbated cutaneous disease (4–6). Given the central role of eosinophilic inflammation in the pathogenesis of NERD, the marked reduction in blood eosinophil counts observed under anti-IL-5/IL-5R therapy likely represents a key mechanism contributing to both improved respiratory tolerance and attenuation of NSAID-induced reactions. In this context, the absence of urticaria among patients with BRs following biologic therapy remains noteworthy. However, previous studies evaluating mepolizumab in chronic spontaneous urticaria have generally reported limited or inconsistent clinical benefit on cutaneous symptoms (33). This suggests that the lack of urticaria observed in our study is unlikely to reflect a direct anti-urticarial effect of anti-IL-5/IL-5R therapy. Instead, it may point toward disease-specific mechanisms related to eosinophilic airway and systemic inflammation in NERD, rather than a primary cutaneous effect. Nevertheless, this hypothesis warrants confirmation in larger, dedicated studies specifically designed to address potential cutaneous outcomes in biologic-treated NERD populations.

The beneficial effects of anti–IL-5/IL-5R biologics on asthma control have been consistently demonstrated in previous studies (34). In our study, patients requiring biologic treatment were initially maintained on higher doses of ICS/LABA. Following treatment, all were able to step down to medium doses, and several voluntarily reduced to low-dose regimens while remaining asymptomatic. This step-down was accompanied by marked improvements in ACT scores, in line with prior reports. Although a reduction to low-dose ICS-LABA is not currently recommended, our findings suggest that some patients may tolerate such regimens without loss of control, a hypothesis that warrants confirmation in future studies. Overall, these observations reinforce the substantial effectiveness of anti–IL-5/IL-5R biologics in improving asthma outcomes.

In line with the findings of the recent meta-analysis by Cai et al., which demonstrated consistent efficacy and safety of dupilumab, omalizumab, mepolizumab, and benralizumab in real-world CRSwNP cohorts, our study also provides supportive evidence for the role of biologics in this setting (35). Although NP grade did not regress in most patients and remained stable in all but two cases, biologic therapy resulted in marked and statistically significant clinical improvement across the study, as reflected by a substantial reduction in SNOT-22 scores and a significant increase in VAS quality-of-life scores. These findings are in line with previous reports indicating that meaningful symptomatic and quality-of-life benefits may occur even in the absence of marked nasal polyp size regression (36, 37). Moreover, patients who underwent concurrent surgery maintained a polyp-free status under biologic treatment, suggesting a possible protective effect against recurrence.

The overall sample size was limited, particularly in the biologic-treated group, and this should be considered when interpreting the findings. However, N-ERD represents a distinct asthma phenotype, affecting approximately 7–10% of adults with asthma and a higher proportion of patients with severe asthma (38). Our cohort represents an even more selected real-world population, comprising patients with NSAID hypersensitivity receiving anti-IL-5/IL-5R therapy who were also willing to undergo repeat ASA provocation testing. Thus, although the sample size limits definitive conclusions, the cohort provides clinically relevant real-world data in a narrowly defined patient population. In addition, the relatively small number of patients receiving benralizumab, which became available in Türkiye later than mepolizumab, precluded a reliable agent-specific comparison. Therefore, mepolizumab- and benralizumab-treated patients were analyzed together as the anti-IL-5/IL-5R-treated group. In addition, given the limited sample size, adjustment for potential confounding factors was not feasible. Therefore, as in all real-world observational studies, residual confounding cannot be excluded. In addition, the biologic-naive comparison group was not fully matched to the biologic-treated group, as patients receiving biologics generally had more severe disease. The biologic-naive group was included primarily to provide a real-world reference for NSAID hypersensitivity outcomes in patients not receiving anti-IL-5/IL-5R therapy and to explore the possibility of spontaneous changes in ASA reactivity. Nevertheless, comparisons between these groups should be interpreted with caution because of the inherent differences in disease severity and the observational design of the study. Although no nasal polyp recurrence was observed among the six patients who underwent ESS during biologic treatment, this finding should be interpreted cautiously because of the small number of patients and because postoperative healing and recurrence were not predefined study outcomes. Furthermore, mechanistic biomarkers were not systematically evaluated in this study, limiting our ability to explore the biological pathways underlying the observed improvement in ASA tolerance. Future studies incorporating serial assessments of relevant inflammatory mediators, including leukotriene E4 (LTE_4_) and prostaglandin D_2_ (PGD_2_), may help clarify the mechanisms associated with anti-IL-5/IL-5R-induced changes in NSAID tolerance.

In addition, increasing the sample size would allow a more robust evaluation of the subgroup that remained ASA-reactive, providing insight into whether biologic therapy exerts differential effects within this population. Finally, our study did not explore the long-term trajectory of tolerance beyond the reported provocation tests; whether patients would continue to maintain their tolerance with ongoing biologic therapy remains an open question. Furthermore, because clear discontinuation criteria for biologics in this patient group are lacking, it also remains uncertain whether tolerance would persist or be lost after cessation of therapy, a limitation we were unable to address in this study and further studies are needed to clarify this issue.

## Conclusion

Anti–IL-5/IL-5R biologics not only improved asthma and sinonasal outcomes but also significantly attenuated NSAID hypersensitivity, reducing ASA provocation positivity from universal to less than one-third of patients. To our knowledge, this is the first study including BRs, with assessments performed as early as the third biologic dose. Even in patients who remained reactive, tolerance improved with higher thresholds and milder symptoms. These results indicate that anti–IL-5 therapy provides benefits extending beyond asthma and nasal polyps, and the parallel reduction in eosinophil burden suggests that attenuation of hypersensitivity to ASA may be linked to decreased eosinophilic inflammation.

## Data Availability

The original contributions presented in the study are included in the article/**Supplementary Material**. Further inquiries can be directed to the corresponding author.
