# Supplementary material for: IL-5/IL-5R blockade enables safe non-steroidal anti-inflammatory agent reintroduction in non-steroidal anti-inflammatory exacerbated respiratory disease
Source: Front Immunol. 2026 Sep 3;17:1950229. doi: 10.3389/fimmu.2026.1950229 (PMC13581757; doi:10.3389/fimmu.2026.1950229)
Supplement: Supplementary file 1 [file Table1.docx]

**Supplement 1.** Characteristics of Patients on Biologic Therapy and Outcomes of ASA Provocation Test

| **Patient ID** | **Age** | **Gender** | **Biologic agent** | **Biologic start age** | **NP grade (before biologic)** | **NP grade (during ASA provocation)** | ***NSAID reaction phenotype*** | ***Cumulative ASA dose at reaction onset (mg)*** | ***Symptom onset time (min)*** | ***n° of biologic received* prior to ASA provocation** | **ASA provocation interval (days after last dose)** | **ASA provocation test** |
| --- | --- | --- | --- | --- | --- | --- | --- | --- | --- | --- | --- | --- |
| **P1** | 50 | M | M | 47 | 3 | 3 | NERD | - | - | 8 | 13 | Negative |
| **P2*** | 34 | M | M | 34 | 4 | 4 | BRs | - | - | 5 | 17 | Negative |
| **P3** | 54 | F | M | 49 | 2 | 2 | NERD | - | - | 57 | 20 | Negative |
| **P4**** | ***31*** | ***F*** | ***B*** | ***31*** | ***Absent NP*** | ***Absent NP*** | ***NERD*** | ***1050*** | ***145*** | ***3*** | ***13*** | ***Nasal symptoms*** |
| **P5** | 23 | F | M | 23 | No NP (post-surgery) | No NP (post-surgery) | NERD | - | - | 3 | 4 | Negative |
| **P6*** | 71 | M | B | 70 | Absent NP | Absent NP | NERD | - | - | 11 | 16 | Negative |
| **P7** | ***30*** | ***F*** | ***M*** | ***27*** | ***2*** | ***1*** | ***NERD*** | ***300*** | ***67*** | ***40*** | ***14*** | ***Dyspnea***, nasal symptoms*** |
| **P8** | 43 | F | M | 39 | No NP (post-surgery) | No NP (post-surgery) | NERD | - | - | 45 | 20 | Negative |
| **P9** | ***25*** | ***M*** | ***M*** | ***25*** | ***2*** | ***1*** | ***NERD*** | ***1050*** | ***137*** | ***3*** | ***3*** | ***Nasal symptoms*** |
| **P10*** | 52 | F | M | 47 | No NP (post-surgery) | No NP (post-surgery) | BRs | - | - | 57 | 5 | Negative |
| **P11** | 49 | M | M | 49 | No NP (post-surgery) | No NP (post-surgery) | NERD | - | - | 3 | 23 | Negative |
| **P12** | ***30*** | ***F*** | ***B*** | ***30*** | ***No NP (post-surgery)*** | ***No NP (post-surgery)*** | ***NERD*** | ***550*** | ***105*** | ***5*** | ***14*** | ***Bronchospasm****, nasal symptoms, conjunctival erythema*** |
| **P13** | 28 | F | M | 25 | 4 | 4 | NERD | - | - | 33 | 30 | Negative |
| **P14** | 39 | M | M | 37 | 3 | 3 | NERD | - | - | 27 | 6 | Negative |

*NSAID: Nonsteroidal Anti-Inflammatory Drug, ASA: Acetylsalicylic Acid, F: Female, M: Male, B: Benralizumab, M: Mepolizumab, NP: Nasal Polyposis, NERD: NSAID-Exacerbated Respiratory Disease, BRs: Blended Reactions*

**Previous omalizumab use*

*** Previous mepolizumab and omalizumab use*

****Although the patient experienced respiratory complaints, spirometry did not reveal any functional decline.*

*****Spirometry revealed a 300 mL (17%) reduction in FEV₁.*
