# Supplementary material for: IL-5/IL-5R blockade enables safe non-steroidal anti-inflammatory agent reintroduction in non-steroidal anti-inflammatory exacerbated respiratory disease
Source: Front Immunol. 2026 Sep 3;17:1950229. doi: 10.3389/fimmu.2026.1950229 (PMC13581757; doi:10.3389/fimmu.2026.1950229)
Supplement: Supplementary file 2 [file Table2.docx]

**Supplement 2.** Clinical characteristics and ASA provocation outcomes in patients without anti–IL-5/IL-5R treatment

| ***Patient ID*** | ***Age*** | ***Gender*** | ***Number of ESS Procedures*** | ***EO during ASA provocation (cells/μL)*** | ***ACT score (during ASA provocation)*** | ***SNOT-22 (during ASA provocation)*** | ***VAS QoL***  ***(during ASA provocation)*** | ***Cumulative ASA dose at reaction onset (mg)*** | ***Symptom onset time (min)*** | ***NP grade (during ASA provocation)*** | ***ICS/LABA dose (during ASA provocation)*** | ***ASA Provocation test*** | ***NSAID Reaction Phenotype Based on ASA Provocation*** |
| --- | --- | --- | --- | --- | --- | --- | --- | --- | --- | --- | --- | --- | --- |
| ***C-1*** | 51 | M | Absent NP | 220 | 25 | 12 | 3 | 550 | 95 | Absent NP | ICS–formoterol as-needed use (reliever) | FEV₁ ↓850 mL (21%), angioedema (eye/lip), systemic urticaria, nasal symptoms | BRs |
| ***C-2*** | 33 | F | Absent NP | 20 | 25 | 8 | 2 | 300 | 72 | Absent NP | ICS–formoterol as-needed use (reliever) | FEV₁ ↓565 mL (20%), angioedema (eye/lip), nasal symptoms, conjunctival erythema | BRs |
| ***C-3*** | 24 | F | 1 | 220 | 22 | 22 | 4 | 1050 | 190 | 2 | Medium | FEV₁ ↓670 mL (25%), nasal symptoms, conjunctival erythema | NERD |
| ***C-4*** | **55** | **M** | **1** | **370** | **25** | **6** | **0** | **-** | **-** | **No NP (post-surgery)** | **Low** | **Negative** | **NERD (Spontaneous remission/improvement within 8 years)** |
| ***C-5*** | 31 | M | 3 | 160 | 25 | 12 | 3 | 550 | 102 | No NP (post-surgery) | ICS–formoterol as-needed use (reliever) | FEV₁ ↓575 mL (19%), nasal symptoms | NERD |
| ***C-6*** | 43 | M | 5 | 470 | 24 | 24 | 3 | 300 | 75 | 1 | Low | FEV₁ ↓680 mL (22%), nasal symptoms | NERD |
| ***C-7*** | 44 | M | 2 | 140 | 25 | 31 | 4 | 1050 | 135 | 2 | ICS–formoterol as-needed use (reliever) | FEV₁ ↓580 mL (20%), nasal symptoms, conjunctival erythema | NERD |
| ***C-8*** | 51 | M | Absent NP | 420 | 25 | 26 | 5 | 1050 | 200 | Absent NP | ICS–formoterol as-needed use (reliever) | FEV₁ ↓2000 mL (37.5%), nasal symptoms, conjunctival erythema, systemic urticaria | BRs |

*ASA: Acetylsalicylic Acid, Il-5: Interleukin-5, ESS: Endoscopic Sinus Surgery, EO: Eosinophil, ACT: Asthma Control Test, SNOT-22: Sino-Nasal Outcome Test-22, VAS: Visual Analog Scale, QoL: Quality of Life, NP: Nasal Polyposis, ICS: Inhaled Corticosteroid, LABA: Long-Acting Beta2-Agonist, NSAID: Nonsteroidal Anti-Inflammatory Drug, F: Female, M: Male, HT: Hypertension, FEV₁: Forced Expiratory Volume in 1 Second, HDM: House Dust Mite, NERD: NSAID-Exacerbated Respiratory Disease, BRs: Blended Reactions, CSU: Chronic Spontaneous Urticaria*
